# Enhanced Esophageal Cancer Detection Using Spectrum‐Aided Vision Enhancer: A Comparative Study of White Light Imaging, Narrow‐Band Imaging, and Algorithmic Narrow‐Band Imaging‐Like Imaging

**DOI:** 10.1002/deo2.70467

**Published:** 2026-11-04

**Authors:** Jer‐Wei Wu, Yao‐Kuang Wang, Arvind Mukundan, Riya Karmakar, Nguyen Ngoc Quynh Nhu, Yaswanth Nagisetti, Wei‐Chih Liao, Ming‐Lun Han, I‐Chen Wu, Hsiang‐Chen Wang

**Affiliations:** ^1^ Division of Endoscopy Department of Integrated Diagnostics & Therapeutics National Taiwan University Hospital Taipei Taiwan; ^2^ Graduate Institute of Clinical Medicine College of Medicine Kaohsiung Medical University Kaohsiung Taiwan; ^3^ Division of Gastroenterology Department of Internal Medicine Kaohsiung Medical University Hospital Kaohsiung Taiwan; ^4^ Department of Medicine Faculty of Medicine College of Medicine Kaohsiung Medical University Kaohsiung Taiwan; ^5^ Department of Mechanical Engineering National Chung Cheng University Chia Yi Taiwan; ^6^ Department of Biomedical Imaging Chennai Institute of Technology Chennai India; ^7^ Department of Computer Science Engineering School of Engineering and Technology Sanjivani University Kopargaon India; ^8^ Department of Integrated Bachelor of Technology School of Engineering and Technology Sanjivani University Kopargaon India; ^9^ Department of Biomedical Engineering International University ‐ Vietnam National University Ho Chi Minh City Vietnam; ^10^ Division of Gastroenterology and Hepatology Department of Internal Medicine National Taiwan University Hospital National Taiwan University College of Medicine Taipei Taiwan; ^11^ Department of Medical Research Dalin Tzu Chi Hospital Buddhist Tzu Chi Medical Foundation Chia Yi Taiwan

**Keywords:** algorithmic imaging, esophageal cancer, narrow‐band imaging, spectrum‐aided vision enhancer (SAVE), white‐light imaging

## Abstract

**Background and Aims:**

Esophageal squamous cell carcinoma (ESCC) detection is crucial for improving survival. While narrow‐band imaging (NBI) improves mucosal visualization, limitations remain. We evaluated the diagnostic performance of the Spectrum‐Aided Vision Enhancer (SAVE), an algorithmic NBI‐like technology reconstructed from white light imaging (WLI).

**Methods:**

We conducted a retrospective, image‐based exploratory feasibility study to descriptively evaluate the diagnostic performance of WLI, NBI, and SAVE. A dataset of 1350 still endoscopic images (450 each for WLI, NBI, and SAVE) across normal mucosa, dysplasia, and ESCC stages from 158 patients was blindly reviewed by three endoscopists with different experience levels (3, 5, and 15 years). Diagnostic metrics included sensitivity, specificity, accuracy, and reading time.

**Results:**

For ESCC, SAVE demonstrated diagnostic performance metrics (sensitivity: 0.81–0.97, specificity: 0.85–0.96) that were numerically similar to WLI and NBI. For dysplasia detection, SAVE had lower observed sensitivity (0.39–0.62) compared to NBI (0.55–0.85) across all observers, representing a clinical limitation for precancerous screening in its current format. Mean per‐image reading times were similar across WLI, NBI, and SAVE.

**Conclusions:**

In this retrospective study, SAVE showed diagnostic potential for ESCC, with observed diagnostic performance metrics numerically similar to those of WLI and NBI in this exploratory dataset, but lacked sensitivity for dysplasia. These findings are hypothesis‐generating and warrant further prospective, multicenter validation to refine the algorithm for early cancer detection.

## 1 Introduction

Esophageal cancer (EC) has emerged as a formidable global health challenge, accounting for approximately 604,100 new diagnoses and 544,076 deaths in 2020 alone, making it a leading cause of oncological mortality worldwide [1, 2, 3]. In high‐incidence regions, such as East Asia and Eastern Africa, esophageal squamous cell carcinoma (ESCC) remains the predominant histological subtype [4]. The clinical significance of early detection is profound; when diagnosed and treated at an early stage, the 5‐year survival rate for EC can reach approximately 90% [1]. However, EC often presents with vague symptoms, leading to late diagnosis. The 5‐year survival rate drops sharply to about 47% at the localized stage and only 5% at the advanced stage [5]. This underscores the critical importance of developing advanced diagnostic tools [4].

Esophageal squamous dysplasia (ESD) is widely recognized as the precursor lesion to ESCC [6]. The progression from dysplasia to invasive carcinoma follows a well‐characterized sequence of histopathological changes [7, 8, 9]. Conventional white light imaging (WLI) remains the primary modality for the initial evaluation of the upper gastrointestinal tract. When integrated with histopathological assessment, WLI facilitates the detection of approximately 80% of early‐stage ECs. Nevertheless, its restricted sensitivity in resolving subtle mucosal abnormalities represents a substantial barrier to the early diagnosis of dysplastic and neoplastic lesions [10]. Consequently, advanced image‐enhanced endoscopy (IEE) modalities, such as narrow‐band imaging (NBI) and hyperspectral imaging (HSI), have emerged to bolster mucosal contrast and refine the differentiation between neoplastic and non‐neoplastic tissues [11, 12, 13].

NBI uses narrow‐band illumination in the blue and green wavelength regions corresponding to hemoglobin absorption peaks, enhancing the visualization of superficial mucosal microvessels and intrapapillary capillary loop patterns [11, 12, 13, 14]. Clinical studies have shown that NBI improves early detection and characterization of neoplastic lesions, reduces false‐negative rates, and minimizes unnecessary random biopsies [14, 15, 16, 17, 18, 19]. HSI captures dense spatial and spectral data over contiguous wavelength bands, showing high sensitivity (85%–95%) and specificity (85%–95%) in distinguishing normal and abnormal tissues in gastrointestinal endoscopy [20, 21, 22, 23, 24, 25, 26, 27, 28]. However, hardware‐based spectral filtering in NBI and HSI requires specialized, expensive optical equipment, which limits their global accessibility. Furthermore, certain lesion morphologies remain difficult to discern even with hardware NBI, and artificial intelligence diagnostic performance remains fundamentally constrained by primary hardware data [10, 29].

To address these limitations, software‐based image‐enhancement tools are being developed as complementary solutions [30, 31, 32, 33, 34]. The Spectrum‐Aided Vision Enhancer (SAVE) computationally estimates spectral reflectance information from conventional RGB WLI images using an RGB‐to‐spectral estimation model. The estimated spectral information is subsequently used to select wavelength bands that generate NBI‐like contrast without requiring dedicated optical filtering hardware [10, 20, 26].

This research was not intended to prove that SAVE is an alternative to existing hardware NBI technology. Rather, the clinical question was whether algorithmic spectral reconstruction was able to create an NBI‐like contrast from conventional WLI images and offer the capability for further image enhancement without the use of dedicated optical filtering equipment. The expected benefits of SAVE may be its use as a software‐based enhancement tool for traditional endoscopy systems, in areas where high‐end IEE equipment is not available, and as a method for post‐acquisition SAVE of WLI data. Thus, the present study was conducted as an exploratory study to define the diagnostic value of the images obtained from the SAVE system in comparison to conventional WLI and NBI.

## 2 Methodology

### 2.1 Dataset and Image Cohort

This research was designed as a pre‐clinical, retrospective, image‐based feasibility study. The evaluation used a curated dataset of 1350 still endoscopic images, equally partitioned into 450 images for each imaging modality (WLI, NBI, and SAVE). Within each modality, images were stratified across three clinical stages: normal mucosa, ESD, and ESCC (150 images per stage). The image cohort was derived from 158 patients, including 49 healthy controls, 56 patients with esophageal dysplasia, and 53 with ESCC. All primary WLI and NBI images were acquired using a high‐definition Olympus CV‐290 endoscopy system (Osaka, Japan) at Kaohsiung Medical University Chung‐Ho Memorial Hospital.

Some other clinicopathological features of ESCC lesions were re‐evaluated. Both superficial and advanced lesions were coded for in ESCC images. The available macroscopic characteristics such as superficial mucosal changes and advanced tumor morphology were recorded, as the morphology may affect the detectability of the lesion by visual inspection. The following characteristics were taken into account when evaluating the diagnostic performance of WLI, NBI, and SAVE: Images were selected based on predetermined standards for image quality and visibility of lesions.

Images that exhibited significant motion artefact, excessive blur, insufficient illumination, inadequate visualization of the lesion, severe image degradation, or insufficient information to allow reliable pathological correlation were discarded. The images that conformed to the quality criteria defined and showed adequate visualization of the target lesion were kept for analysis. No further selection based on the supposed difficulty in diagnosis of the lesion was carried out

### 2.2 Computational Spectral Estimation and SAVE Image Generation

SAVE images were computationally generated from conventional RGB WLI images using a spectral estimation framework. The algorithm recovers estimated spectral reflectance and processes it through wavelength selection emphasizing hemoglobin absorption peaks (∼450 and ∼540 nm) to generate NBI‐like contrast (Videos S1 and S2). No hyperspectral camera or direct hyperspectral image acquisition was involved. The technical workflow of model training, calibration using the Macbeth Color Checker, and Cauchy–Lorentz modeling with dual annealing is described in detail in Material S1 (Figure 1A and Figures S1–S3).

**FIGURE 1:**
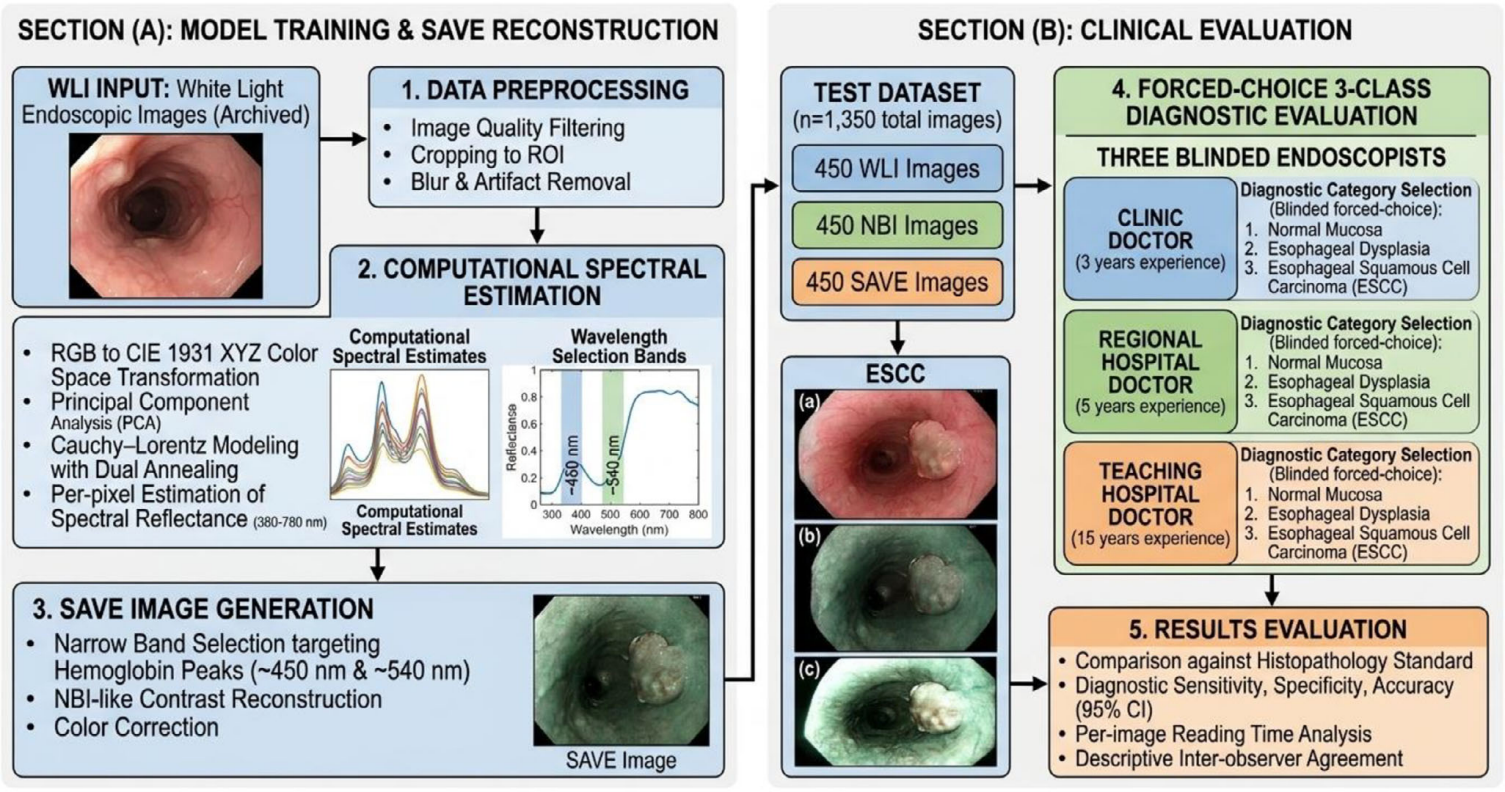
Experimental flowchart. (A) Model training: WLI images are first color‐corrected and illumination‐compensated, converted to the CIE 1931 XYZ space, and calibrated via the correction matrix [C] using [XYZSpectrum] × pinv([V]). Principal components are extracted by PCA, and a regression‐derived transform [M] is obtained to compute [XYZCorrect] = [C] × [V], accounting for nonlinear response and dark current. A computational RGB‐to‐spectral estimation step is then applied using the regression‐derived transform [M], followed by per‐pixel estimation of spectral reflectance over the 380–780 nm range. Band selection emphasizes hemoglobin absorption regions (∼450 and ∼540 nm). Spectral shape calibration against Olympus narrow‐band imaging (NBI) is optimized using the Macbeth Color Checker and Cauchy–Lorentz modeling with dual annealing, while SSIM, entropy, and PSNR guide denoising and fidelity. (B) Model and physician evaluation: The finalized Spectrum‐Aided Vision Enhancer (SAVE) images, together with original white‐light imaging (WLI) and NBI (450 images per modality; 150 per class: normal, dysplasia, ESCC), are blindly reviewed by three endoscopists with different experience levels (3, 5, and 15 years). Using pathology/clinical standards as reference, sensitivity, specificity, accuracy, PPV, NPV, and per‐image reading time are computed across modalities and stages, with inter‐observer comparisons to assess robustness and clinical utility. (*Note*: A detailed description of the SAVE method is provided in Material S1).

### 2.3 Study Design and Ethical Considerations

The study protocol adhered to the ethical principles of the 1964 Declaration of Helsinki and was approved by the Institutional Review Boards of Kaohsiung Medical University Chung‐Ho Memorial Hospital (KMUHIRB‐E(I)‐20230211; May 18, 2023) and National Taiwan University Hospital (No. 202301103RIND; February 15, 2024). Due to the retrospective, anonymized design, the requirement for written informed consent was waived. Multiple images from the same patient were analyzed as independent observations, and no dataset partitioning into training, validation, or testing subsets was performed.

### 2.4 Reference Standard, Blinding, and Image Handling

The reference standard for lesion classification was histopathological diagnosis obtained from endoscopic biopsy or resection specimens. Image‐lesion correspondence was established by comparing endoscopic images with pathology reports and lesion locations. Images with poor quality, motion artifacts, inadequate illumination, or ambiguous correspondence were excluded. Anonymous evaluation was carried out in a randomized sequence. Three endoscopists with different experience levels (3, 5, and 15 years) independently reviewed the images, blinded to patient clinical history, pathological outcomes, and other readers' evaluations (Figure 1B).

### 2.5 Statistical Analysis

Diagnostic performance was assessed at the image level. Sensitivity and specificity were calculated against the histopathological reference standard, and 95% confidence intervals (95% CIs) were estimated using the Wilson binomial proportion method based on sample counts. Accuracy, positive predictive value (PPV), and negative predictive value (NPV) were estimated as point statistics. Because the study was exploratory, no a priori sample size calculation, statistical power calculation, or inferential hypothesis testing (e.g., superiority or equivalence testing) was conducted. No statistical adjustment for intra‐patient clustering was performed, and the reported confidence intervals characterize sample‐level variation only.

The number of images contributed by each individual patient was not known for the retrospective data set due to lack of patient‐to‐image linkage information. Multiple images could then have been taken from the same patient, and these images were considered as separate observations in the image‐level analysis. Therefore, it was not possible to determine or control for within‐patient clustering.

## 3 Results

### 3.1 Normal Mucosa Category

In the normal mucosa category (Figure 2), all three imaging modalities showed high observed sensitivity among the different observers: WLI ranged from 0.99 to 1.00 (95% CI: 0.95–1.00), NBI from 0.93 to 0.99 (95% CI: 0.88–1.00), and SAVE from 0.94 to 0.97 (95% CI: 0.89–0.99) (Table 1). The observed specificity values were more dispersed: WLI was 0.75–0.86, NBI was 0.72–0.93, and SAVE was 0.74–0.90. Accuracies, PPVs, and NPVs varied across observers, representing disparities in image classification rather than verified diagnostic cases. Mean per‐image reading times were similar across modalities: 2.1–2.7 s for WLI, 1.7–3.4 s for NBI, and 1.6–3.0 s for SAVE.

**FIGURE 2:**
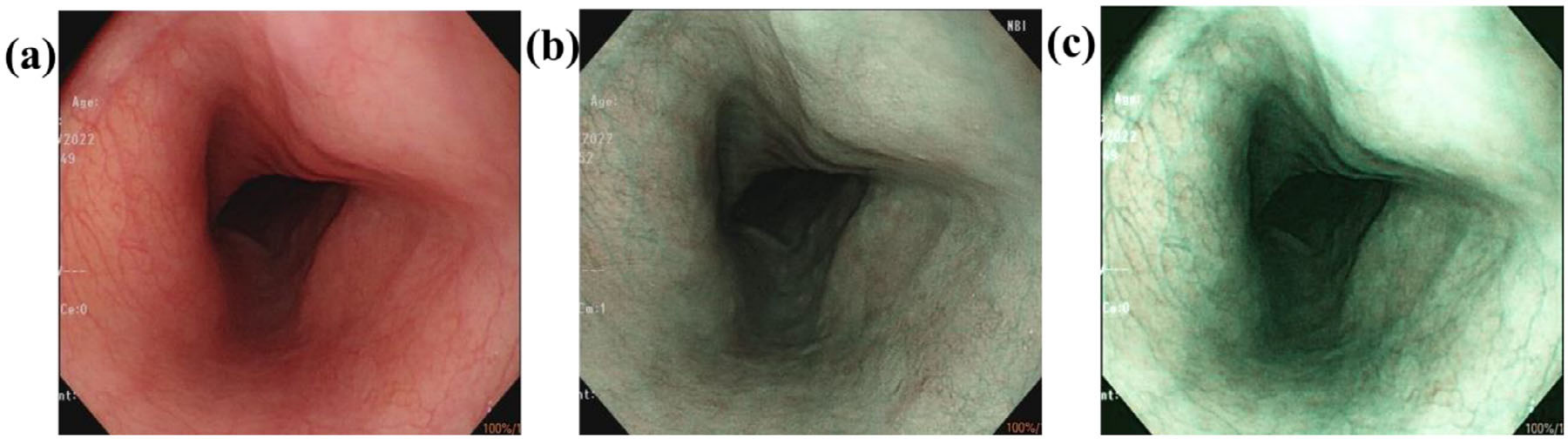
Representative normal esophageal images obtained using (a) white‐light imaging (WLI), (b) narrow‐band imaging (NBI), and (c) Spectrum‐Aided Vision Enhancer (SAVE).

**TABLE 1:** Diagnostic performance of white‐light imaging (WLI), narrow‐band imaging (NBI), and Spectrum‐Aided Vision Enhancer (SAVE) across normal, dysplasia, and esophageal squamous cell carcinoma (ESCC) stages.

Type	Observers	Image type	Sensitivity	Sensitivity 95% CI	Specificity	Specificity 95% CI	Accuracy	PPV	NPV	Time(s/image)
Normal	Regional hospital doctor	WLI	0.99	0.95–1.00	0.86	0.81–0.90	0.9	0.78	1.00	2.2
		NBI	0.93	0.88–0.96	0.89	0.85–0.92	0.9	0.81	0.96	2.6
		SAVE	0.94	0.89–0.97	0.90	0.86–0.93	0.91	0.82	0.97	2.2
	Clinic doctor	WLI	1.00	0.98–1.00	0.75	0.70–0.80	0.83	0.67	1.00	2.1
		NBI	0.99	0.95–1.00	0.72	0.67–0.77	0.81	0.64	1.00	1.7
		SAVE	0.97	0.93–0.99	0.74	0.69–0.79	0.82	0.65	0.98	1.6
	Teaching hospital doctor	WLI	0.99	0.95–1.00	0.80	0.75–0.84	0.86	0.71	1.00	2.7
		NBI	0.94	0.89–0.97	0.93	0.90–0.96	0.93	0.87	0.97	3.4
		SAVE	0.97	0.93–0.99	0.85	0.80–0.89	0.89	0.76	0.98	3.0
Esophageal dysplasia	Regional hospital doctor	WLI	0.57	0.49–0.65	0.98	0.95–0.99	0.84	0.93	0.82	3.6
		NBI	0.69	0.61–0.76	0.80	0.75–0.84	0.76	0.63	0.84	3.4
		SAVE	0.51	0.43–0.59	0.96	0.93–0.98	0.81	0.87	0.80	3.4
	Clinic doctor	WLI	0.40	0.33–0.48	0.98	0.95–0.99	0.79	0.91	0.77	2.90
		NBI	0.55	0.47–0.63	0.81	0.76–0.85	0.72	0.59	0.78	2.6
		SAVE	0.39	0.32–0.47	0.91	0.87–0.94	0.74	0.69	0.75	2.2
	Teaching hospital doctor	WLI	0.59	0.51–0.67	0.96	0.93–0.98	0.84	0.87	0.82	4.70
		NBI	0.85	0.78–0.90	0.77	0.72–0.82	0.80	0.65	0.91	3.9
		SAVE	0.62	0.54–0.69	0.94	0.91–0.96	0.83	0.84	0.83	4.0
ESCC	Regional hospital doctor	WLI	0.94	0.89–0.97	0.92	0.88–0.95	0.93	0.85	0.97	1.5
		NBI	0.63	0.55–0.70	0.94	0.91–0.96	0.84	0.84	0.84	2.0
		SAVE	0.97	0.93–0.99	0.85	0.80–0.89	0.89	0.76	0.98	1.6
	Clinic doctor	WLI	0.94	0.89–0.97	0.94	0.91–0.96	0.94	0.89	0.97	2.6
		NBI	0.50	0.42–0.58	0.99	0.97–1.00	0.83	0.96	0.80	2.4
		SAVE	0.81	0.74–0.86	0.94	0.91–0.96	0.90	0.87	0.91	2.0
	Teaching hospital doctor	WLI	0.91	0.85–0.95	0.99	0.97–1.00	0.96	0.98	0.96	3.20
		NBI	0.57	0.49–0.65	0.98	0.95–0.99	0.84	0.93	0.82	4.2
		SAVE	0.91	0.85–0.95	0.96	0.93–0.98	0.94	0.92	0.96	3.1

CI, confidence interval; ESCC, esophageal squamous cell carcinoma; NBI, narrow‐band imaging; SAVE, Spectrum‐Aided Vision Enhancer; WLI, white‐light imaging. Note on Observers: The three evaluators represent endoscopists at different practice settings and clinical experience levels: Clinic doctor (3 years of experience), Regional hospital doctor (5 years of experience), and Teaching hospital doctor (15 years of experience). The 95% confidence intervals for sensitivity and specificity were calculated using the Wilson binomial proportion method. Sensitivity confidence intervals were calculated using disease‐positive images (*n* = 150), whereas specificity confidence intervals were calculated using disease‐negative images (*n* = 300)

### 3.2 Esophageal Squamous Dysplasia Category

For esophageal dysplasia (Figure 3), WLI reported low observed sensitivity levels ranging from 0.40 to 0.59 (95% CI: 0.33–0.67), whereas specificity levels were high at 0.96–0.98 (95% CI: 0.93–0.99) (Table 1). NBI demonstrated higher observed sensitivity values compared with WLI, ranging from 0.55 to 0.85 (95% CI: 0.47–0.90), with specificity levels of 0.77–0.81 (95% CI: 0.72–0.85). SAVE showed an observed sensitivity of 0.39 to 0.62 (95% CI: 0.32–0.69) and specificity of 0.91 to 0.96 (95% CI: 0.87–0.98). Across all observers, SAVE demonstrated lower sensitivity than NBI, identifying fewer dysplastic lesions. This represents a clinically significant limitation of SAVE in its current format for precancerous screening. Mean per‐image reading times were 2.9–4.7 s in WLI, 2.6–3.9 s in NBI, and 2.2–4.0 s in SAVE.

**FIGURE 3:**
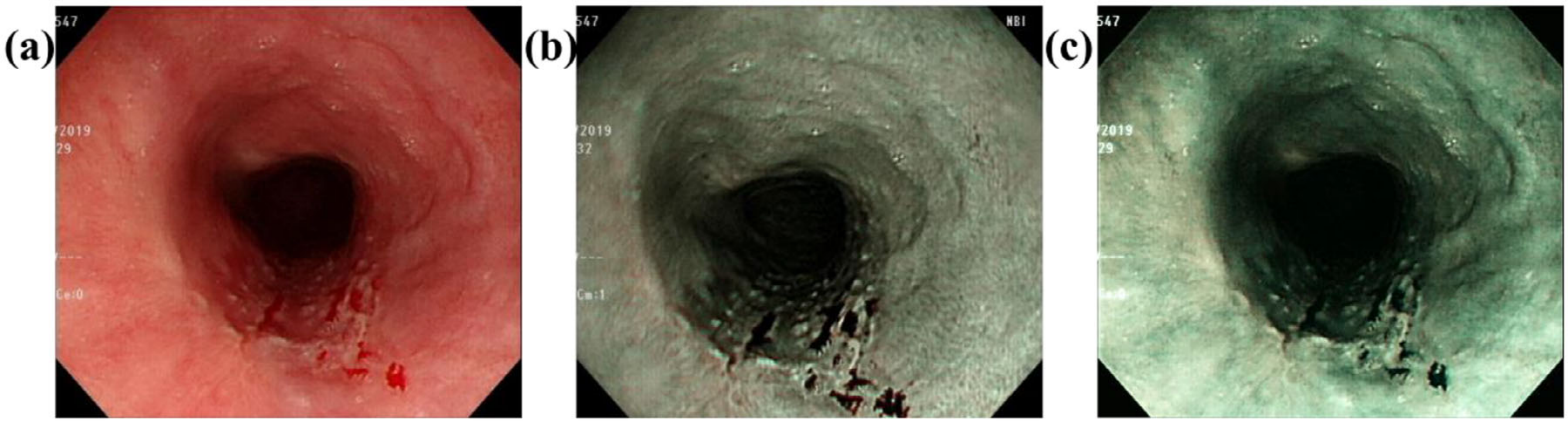
Representative corresponding images of esophageal dysplasia obtained using three imaging modalities from the same lesion region: (a) white‐light imaging (WLI), (b) narrow‐band imaging (NBI), and (c) Spectrum‐Aided Vision Enhancer (SAVE).

### 3.3 ESCC Category

In the ESCC category (Figure 4), observed sensitivity and specificity values across all observers were high in all three modalities. WLI sensitivity ranged from 0.91 to 0.94 (95% CI: 0.85–0.97) and specificity from 0.92 to 0.99 (95% CI: 0.88–1.00) (Table 1). NBI sensitivity ranged from 0.50 to 0.63 (95% CI: 0.42–0.70) and specificity from 0.94 to 0.99 (95% CI: 0.91–1.00). SAVE demonstrated an observed sensitivity of 0.81 to 0.97 (95% CI: 0.74–0.99) and specificity of 0.85 to 0.96 (95% CI: 0.80–0.98). Average per‐image reading times were 1.5–3.2 s in WLI, 2.0–4.2 s in NBI, and 1.6–3.1 s in SAVE. No formal statistical comparisons (superiority, non‐inferiority, or equivalence testing) were performed, and predictive values (PPV/NPV) must be interpreted with caution due to the artificially prevalence‐balanced dataset.

**FIGURE 4:**
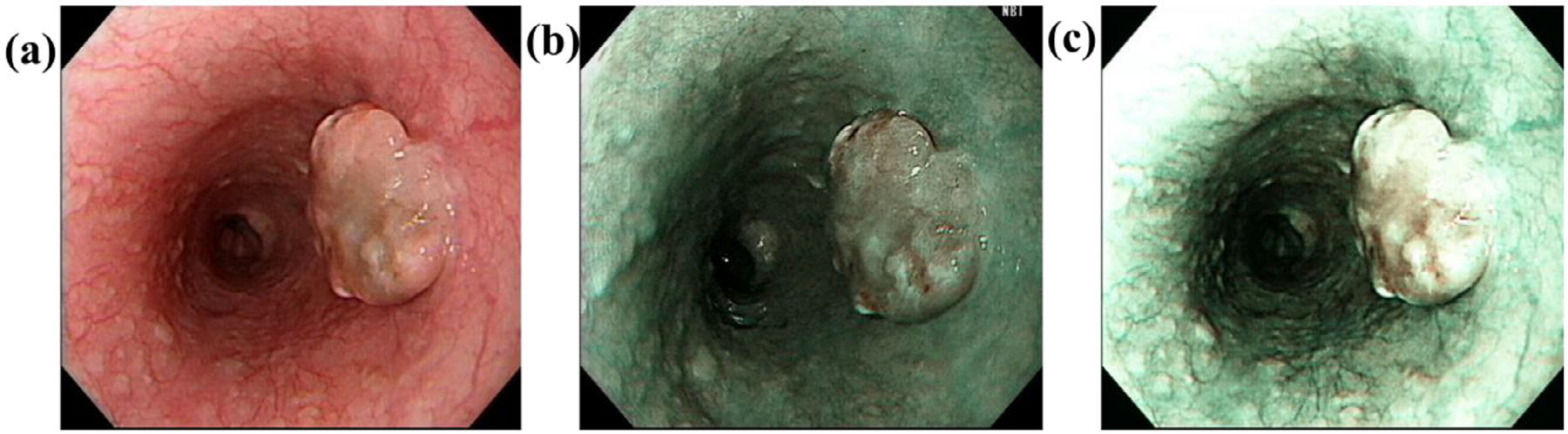
Representative corresponding images of esophageal squamous cell carcinoma (ESCC) obtained using three imaging modalities: (a) white‐light imaging (WLI), (b) narrow‐band imaging (NBI), and (c) Spectrum‐Aided Vision Enhancer (SAVE).

## 4 Discussion

NBI is a technique that is already established and is the most well‐known IEE technology, which has the potential to enhance visualization of the mucosal and vascular structures, but it is dependent on optical hardware on the endoscopic platform. A conceptual difference between SAVE and conventional WLI input is that the enhanced images are algorithmically reconstructed from the conventional WLI input without optical filters. Thus, SAVE is not recommended to replace NBI, but rather as an additional software‐based image enhancement solution that could broaden the availability of image enhancement in systems that do not have specific NBI capability.

This retrospective comparative study evaluated the diagnostic performance of conventional WLI, hardware‐based NBI, and the algorithm‐driven SAVE in EC and premalignant lesion detection. The primary objective was not to establish definitive statistical superiority or clinical non‐inferiority, but to investigate whether the diagnostic patterns of SAVE align with existing clinical modalities.

In ESCC detection, SAVE demonstrated numerically higher observed sensitivity and specificity than NBI in this still‐image dataset. These findings align with previous literature suggesting that spectral reconstruction can improve the visualization of microvascular and mucosal irregularities by amplifying hemoglobin‐related contrast [25, 27]. However, in the detection of squamous dysplasia, SAVE demonstrated lower sensitivity compared with NBI. Since sensitivity is the primary clinical priority for early dysplasia screening, this lower sensitivity represents a critical limitation of SAVE in its current form.

This performance gap can be attributed to SAVE's computational logic. Unlike hardware‐based NBI or HSI, SAVE does not acquire high‐fidelity spectral data directly but instead reconstructs an estimated spectral reflectance from conventional RGB WLI images [10, 20, 26, 28]. Consequently, the reconstructed spectrum depends heavily on the accuracy of the algorithm and cannot perfectly replicate the spectral details of physical optical filters, resulting in lower sensitivity to subtle dysplastic mucosal changes [20, 26]. FICE has been tested in patients with suspicious esophageal lesions. The detection rate of early ESCC with FICE was 92.6%, which was not significantly different from 88.9% on Lugol chromoendoscopy, and magnifying FICE was 96.3%, compared with 92.6% on magnifying Lugol chromoendoscopy (not statistically significant) [35].

There is also some prospective evidence for i‐SCAN. As part of a prospective non‐inferiority study, 356 patients underwent a study of esophageal neoplasia; 42 of these patients had 47 histologically proven esophageal neoplasias. i‐SCAN was non‐inferior to iodine chromoendoscopy for detection of esophageal squamous neoplasia, with a 10.4% detection rate compared to 12.9% with Lugol chromoendoscopy. This study, however, was carried out at a single center [36].

The evidence for TXI is more limited and has mostly examined image visibility and contrast in lesions, not the ability to predict cancer rates. The study included 59 squamous‐cell‐carcinoma‐suspicious lesions in 30 patients; TXI mode 1 achieved higher differences in color between the lesions and the background than WLI, and 62.5% of the lesions were more visible based on the responses of over half of the endoscopists participating in the study. A later retrospective analysis of 20 ESCC lesions also found that with iodine staining, TXI had a greater color contrast and visibility than WLI. From these findings, the authors conclude that TXI can enhance lesion visualization, but this needs to be confirmed by future studies showing a better ability to detect esophageal neoplasia [37].

While these techniques, FICE, i‐SCAN and TXI, have the potential to provide improved visualization and/or detection of esophageal neoplasia, the available data are currently varied and do not confirm a clear superiority over existing optical IEE. The technologies should thus be seen as complementary rather than a replacement for NBI. Unlike these technologies, SAVE also involves computational image enhancement, but, in addition to making NBI‐like contrast, it reconstructs estimated spectral information from traditional RGB WLI images based on an RGB‐to‐spectral reconstruction framework.

Methodological limitations must be acknowledged. First, this was a retrospective, single‐center, image‐based study with a moderate sample size, making performance estimates subject to random variation [28]. Secondly, the analyses were conducted at the image level, without taking into account within‐patient or within‐lesion clustering adjustments. It was not possible to know how many images were contributed by each individual patient in the retrospective dataset because information on patients' linkage to images was not available. Thus, a statistical assessment of possible correlations between several images from the same patient was not possible. This restriction could lead to an underestimation of uncertainty and over‐stable estimates of diagnostic performance. Future prospective studies should continue to link the images to the patients and should employ the proper patient‐level or clustered statistical analyses [24, 38]. Third, the lower observed sensitivity of NBI for ESCC compared to historical clinical trials is likely a consequence of still‐image evaluation, which lacks the dynamic visualization, magnification, and angle adjustment of live endoscopy [10, 39, 40]. Fourth, the present study evaluates only preselected still images. While SAVE aims to provide software‐based image enhancement, its practical utility during routine endoscopy necessitates real‐time image conversion. At present, real‐time video processing capabilities for the SAVE system have not yet been established. Achieving real‐time processing—including optimized processing speed, low latency, and adequate frame rates—is an important limitation of the current study and serves as a critical prerequisite for future clinical implementation. Fifth, macroscopic lesion‐level data, such as the standardized classification of lesions and depth of invasion, were not always available in the retrospective image dataset. Thus, a statistically sound morphology‐stratified diagnostic performance analysis could not be carried out. There were several factors that could have affected the diagnostic performance of NBI. In the present study, still images, as opposed to live endoscopic examinations, were evaluated. While images that were excessively blurred, had a motion artifact, were poorly illuminated, or poorly visualized for lesions were excluded, there was no standardized quantitative NBI image‐quality score. The possible influence of lesion morphology and image quality on reader performance, therefore, could not be quantitatively evaluated. These should be taken into consideration in future prospective studies with a predetermined image‐quality scoring, a standardized classification of the lesion, and a real‐time evaluation of the image quality by the endoscopists. Finally, the algorithm was calibrated specifically to Olympus endoscopy platforms, and its generalizability remains to be verified.

Future prospective, multicenter studies should incorporate larger, representative patient cohorts and utilize patient‐level clustering‐adjusted statistical methods, such as generalized estimating equations or mixed‐effects models [30, 32, 38, 41]. Furthermore, combining SAVE with machine learning frameworks (e.g., YOLO) and extending the spectral range to near‐infrared wavelengths could improve early dysplasia detection and reduce inter‐observer variability [26, 30, 32, 38].

## 5 Conclusion

In conclusion, this retrospective exploratory study demonstrates the technical feasibility of using the software‐based SAVE algorithm to reconstruct NBI‐like contrast from conventional WLI images. Descriptively, SAVE showed diagnostic potential for ESCC, with observed diagnostic performance metrics numerically similar to those of established imaging modalities in this exploratory dataset. However, its lower sensitivity for detecting squamous dysplasia represents a significant clinical limitation in its current form. These findings are hypothesis‐generating and support further prospective, patient‐level, multicenter trials to refine the algorithmic spectral reconstruction and evaluate its integration into clinical workflows.

## Author Contributions

**Conceptualization**: J.‐W.W., Y.‐K.W., and H.‐C.W.; **Methodology**: J.‐W.W., Y.‐K.W., and H.‐C.W.; **Software**: A.M., R.K., Y.N., and N.N.Q.N.; Validation: A.M., R.K., and N.N.Q.N.; **Formal analysis**: J.‐W.W., Y.‐K.W., A.M., and R.K.; **Investigation**: J.‐W.W., Y.‐K.W., W.‐C.L., M.‐L.H., and I.‐C.W.; **Resources**: W.‐C.L., M.‐L.H., I.‐C.W., and H.‐C.W.; **Data curation**: J.‐W.W. and Y.‐K.W.; **Writing – original draft preparation**: J.‐W.W. and Y.‐K.W.; **Writing – review & editing**: H.‐C.W.; **Visualization**: A.M., R.K., and Y.N.; **Supervision, project administration, and funding acquisition**: H.‐C.W.

## Funding

This research was supported by the National Science and Technology Council, the Republic of China, under the grants NSTC 113‐2221‐E‐194‐011‐MY3 and 113‐2221‐E‐037‐005‐MY2.

## Ethics Statement

This study was conducted in accordance with the Declaration of Helsinki and was approved by the Institutional Review Board of National Taiwan University Hospital (Approval No. 202301103RIND) and the Institutional Review Board of Kaohsiung Medical University Chung‐Ho Memorial Hospital (Approval No. KMUHIRB‐E(I)‐20230211).

## Consent

Written informed consent was waived in this study because of the retrospective, anonymized nature of the study design.

## Conflicts of Interest

The authors declare no conflicts of interest.

## Supporting information




**FIGURE S1** Normalized illumination spectra of Olympus white light imaging (WLI) and the algorithmically reconstructed SAVE.
**FIGURE S2** Computational spectral estimation from RGB WLI images. Wavelength (nm) is represented on the x‐axis, and normalized intensity (arbitrary units) is represented on the y‐axis. The displayed SAVE spectra are computational estimates derived from RGB WLI data and are provided for descriptive comparison; they do not represent directly acquired hyperspectral measurements.
**FIGURE S3** Olympus white light imaging (WLI) White light illumination spectrum where wavelength bands are selected by the SAVE.

**VIDEO S1** Conventional endoscopic video of the esophagus captured using Olympus White Light Imaging (WLI), showing the baseline visualization of mucosal surfaces and vascular patterns.

**VIDEO S2** Reconstructed endoscopic video of the same esophageal region using the SAVE algorithm, demonstrating enhanced NBI‐like contrast and improved visualization of superficial microvascular structures.

## Data Availability

The data presented in this study are available in this article upon request to the corresponding author (Hsiang‐Chen Wang).
