# Supplementary material for: Enhanced Esophageal Cancer Detection Using Spectrum‐Aided Vision Enhancer: A Comparative Study of White Light Imaging, Narrow‐Band Imaging, and Algorithmic Narrow‐Band Imaging‐Like Imaging
Source: DEN Open. 2026 Nov 4;7(1):e70467. doi: 10.1002/deo2.70467 (PMC13733652; doi:10.1002/deo2.70467)
Supplement: Supplementary file 1 [file DEO2-7-e70467-s001.docx]

Enhanced Esophageal Cancer Detection Using SAVE: A Comparative Study of WLI, NBI, and Algorithmic NBI-Like Imaging (with video): Supplementary Document

**Supplementary Material 1: Technical Framework of the Spectrum-Aided Vision Enhancer (SAVE)**

The SAVE does not take hyperspectral images directly. Instead, it estimates the spectral reflectance information from typical RGB (white-light) images by using a computational RGB-to-spectral estimation model. The reconstructed spectra are then subjected to the process of wavelength selection to create NBI-like contrast. Thus, in this study, the term 'HSI' does not mean that the image is acquired via hardware but rather the computational reconstruction model.

The SAVE technique converts WLI images into SAVE images resembling Olympus NBI by combining computational spectral estimation with band selection to enhance contrast between normal and cancerous tissues, offering a cost-effective alternative to hardware-based NBI. The process begins with color conversion and correction, addressing inaccuracies in RGB values caused by camera limitations and varying light conditions. The SAVE algorithm utilizes a specific band selection mechanism to reconstruct the narrow-band-like contrast from the original WLI data. As shown in Figure S1, the selection process emphasizes the spectral intervals that align with the hemoglobin absorption peaks, specifically focusing on the 450 nm and 540 nm regions. This targeted spectral reconstruction allows the software to enhance the visualization of superficial microvascular structures without the need for additional hardware filters. Using VIS-HIS, RGB images are transformed into estimated spectral representations by associating light reflectance data with each pixel. A Macbeth color checker, featuring 6 gray shades and 18 natural colors, is used for calibration. The endoscope captures this palette, comparing actual color reflections with recorded RGB values to detect discrepancies. RGB values are then converted to the CIE 1931 XYZ color space for better alignment with human perception. To correct distortions like non-linear response and color inaccuracies, equation (1) applies a correction matrix [C], which uses the pseudo-inverse of the transformation matrix [pinv([V])] to adjust for illumination and sensor response. This ensures accurate mapping of RGB values to the XYZ color space, improving image fidelity.

[C] = [XYZ_Spectrum_] × pinv([V])	(1)

After learning the XYZ values, equation 2 calibrates a process to further correct environmental and non-linear factors of the camera for high accuracy in the true spectral reflectance of muscle. This process reduces color deviation between the image and real tissue. The spectrometer provided the initial *XYZSpectrum* values, where the Y value was proportional to the brightness. After normalization, the Y value was used in order to calculate the luminance k on which the *XYZSpectrum* acquisition was based. On the basis of performing PCA and multiple regression analysis, PS is computed in determining the principal components, while M is determined as the conversion matrix. Since the RMSE between *XYZSpectrum* and *XYZCorrect* was 0.5355, it needed further correction. The final transformation matrix V was then calculated having elements such as *VColor*, X, Y and Z combinations, nonlinear response and dark current corrections.

[XYZ_Correct_] = [C] × [V] (2)

Once it is transformed, the following procedure will be spectral mapping as shown in Equation 3. In this equation, M stands for the transformation matrix that maps the corrected XYZ values to the reflectance spectrum. The score matrix contains the principal components, which are obtained by PCA of the reflectance data. Since PCA retains the most important components, it reduces the complexity of the data—it focuses on the most important principal components which retain most of the variance. It extracts six principal components that contain 99.64% of the spectral information needed for computational spectral estimation. It also helps in the removal of irrelevant data, optimizes image processing, and makes sure that the system maps the XYZ values correctly to their true reflectance spectra with the minimum loss of information. The spectrum at each image pixel is generated by the use of Equation 4.

[M] = [Score] × pinv([V_Color_])	(3)

[SSpectrum]_380~780nm_ = [EV][M][V_Color_]	(4)

The reconstructed spectral data (S_Spectrum_) are synthesized by applying eigenvector matrices (EV) and transformation matrices (M)—both derived from the Principal Component Analysis (PCA) process—to the corrected XYZ coordinates. This mathematical framework enables the pixel-level computational estimation of spectral data from standard XYZ color space inputs. To ensure clinical cross-modality consistency, the SAVE illumination spectrum is specifically optimized to align with the hardware requirements of the Olympus endoscopy system. This optimization minimizes color discrepancies between simulated and hardware-generated NBI images through a calibration process utilizing the Macbeth Color Checker palette. A primary focus is placed on the hemoglobin absorption peaks at 450 nm and 540 nm, ensuring that the algorithmic contrast enhancement accurately mirrors the physiological visualization provided by Olympus NBI.

Following initial spectral matching, the system undergoes further refinement to mitigate residual color variances. These differences are primarily attributed to three interrelated factors: the illumination spectrum, color matching functions, and reflectance spectra. The calibration protocol involves two critical evaluative steps: The CIEDE 2000 formula is employed to calculate the color difference between the original WLI and the synthesized SAVE images. Significant discrepancies arising from device-specific spectral contrasts are systematically assessed using a 24-color Macbeth Color Checker, as illustrated in the experimental setup (Figure S2). NBI uses wavelengths of 450 nm (blue) and 540 nm (green), but Olympus NBI images also include orange and brown hues, indicating the use of additional wavelengths. The actual wavelength intervals are (442–455), (575–591), and (639–664), as shown in Figure S3. Despite similar intensities, significant differences exist in the 450 and 540 nm spectra, necessitating spectral calibration within this range. Calibration is performed using the Cauchy-Lorentz distribution (Equation 5), with further optimization of Olympus NBI images using the 24-color Macbeth Color Checker. Dual annealing optimization, combining simulated annealing algorithms with local search, reduces the average CIEDE 2000 color difference to 5.36, considered negligible. Additional metrics, including the Structural, Similarity Index Metric (SSIM), entropy, and PSNR, ensure the SAVE-generated images are denoised while maintaining diagnostic accuracy. Diagnostic performance was characterized using standard descriptive metrics, including sensitivity, specificity, accuracy, positive predictive value (PPV), and negative predictive value (NPV). In the absence of formal statistical hypothesis testing or paired confidence interval estimations, the comparative evaluations among WLI, NBI, and SAVE remain strictly exploratory. Accordingly, these findings are intended to provide a preliminary characterization of diagnostic trends and should be interpreted as hypothesis-generating rather than inferential evidence of clinical equivalence or superiority.

$f\left( x;x_{0},\gamma\right)=\frac{1}{\pi\gamma\left[ 1+\left( \frac{x-x_{0}}{\gamma} \right)^{2} \right]}=\frac{1}{\pi}\left[ \frac{\gamma}{\left( x-x_{0} \right)^{2}+y^{2}} \right]$	(5)

***Figure S1. Normalized illumination spectra of Olympus white light imaging (WLI) and the algorithmically reconstructed Spectrum-Aided Vision Enhancer (SAVE).***

***Figure S2. Computational spectral estimation algorithm. Wavelength (nm) is represented by the x-axis, and normalized intensity (arbitrary units) is represented by the y-axis. SAVE spectra are solely displayed for descriptive comparison and are computed from WLI data.***

***Figure S3. Olympus white light imaging (WLI) White light illumination spectrum where wavelength bands are selected by the Spectrum-Aided Vision Enhancer (SAVE).***
